# Does Environment Filtering or Seed Limitation Determine Post-fire Forest Recovery Patterns in Boreal Larch Forests?

**DOI:** 10.3389/fpls.2018.01318

**Published:** 2018-09-11

**Authors:** Wen H. Cai, Zhihua Liu, Yuan Z. Yang, Jian Yang

**Affiliations:** ^1^CAS Key Laboratory of Forest Ecology and Management, Institute of Applied Ecology, Chinese Academy of Sciences, Shenyang, China; ^2^Key Laboratory of Environment Change and Resources Use in Beibu Gulf, Guangxi Teachers Education University, Ministry of Education, Nanning, China; ^3^Guangxi Key Laboratory of Earth Surface Processes and Intelligent Simulation, Guangxi Teachers Education University, Nanning, China; ^4^Department of Forestry and Natural Resources, Thomas Poe Cooper Building, University of Kentucky, Lexington, KY, United States

**Keywords:** fire disturbance, climate change, environmental filtering, seed availability, forest recovery, boosted regression tree analysis

## Abstract

Wildfire is a primary natural disturbance in boreal forests, and post-fire vegetation recovery rate influences carbon, water, and energy exchange between the land and atmosphere in the region. Seed availability and environmental filtering are two important determinants in regulating post-fire vegetation recovery in boreal forests. Quantifying how these determinants change over time is helpful for understanding post-fire forest successional trajectory. Time series of remote sensing data offer considerable potential in monitoring the trajectory of post-fire vegetation recovery dynamics beyond current field surveys about structural attributes, which generally lack a temporal perspective across large burned areas. We used a time series of the normalized difference vegetation index (NDVI) and normalized difference shortwave infrared reflectance index (NDSWIR) derived from Landsat images to investigate post-fire dynamics in a Chinese boreal larch forest. An adjacent, unburned patch of a similar forest type and environmental conditions was selected as a control to separate interannual fluctuation in NDVI and NDSWIR caused by climate from changes due to wildfire. Temporal anomalies in NDVI and NDSWIR showed that more than 10 years were needed for ecosystems to recover to a pre-fire state. The boosted regression tree analysis showed that fire severity exerted a persistent, dominant influence on vegetation recovery during the early post-fire successional stage and explained more than 60% of variation in vegetation recovery, whereas distance to the nearest unburned area and environmental conditions exhibited a relatively small influence. This result indicated that the legacy effects of fire disturbance, which control seed availability for tree recruitment, would persist for decades. The influence of environmental filtering could increase with succession and could mitigate the initial heterogeneity in recovery caused by wildfire.

## Introduction

Wildfire is the primary natural disturbance in boreal forest ecosystems and plays a key role in regulating the carbon, water and energy exchange between the land and atmosphere via its influence on ecosystem structure and function in the region (Harden et al., 2000; Carcaillet et al., 2001; Conard et al., 2002; Kashian et al., 2006; Flannigan et al., 2009; Young et al., 2016). Due to climate-induced increases in fire frequency, severity and size, a possible shift in forest composition from conifer (e.g., *Picea* spp. and *Abies* spp.) toward broadleaf (e.g., *Betula* spp. and *Populus* spp.) species may significantly alter the land surface radiative budget (Amiro et al., 2006; Randerson et al., 2006) and initiate a positive/negative feedback to climate (Balshi et al., 2009; Goulden et al., 2011; Shuman et al., 2011). Therefore, understanding the mechanisms that determine post-fire forest recovery is critical to predicting the potential response of forest ecosystems and its climate feedback in boreal regions.

Previous studies have identified that seed limitation and environmental filtering are two key processes that control post-fire species composition and subsequent successional dynamics in boreal ecosystems (Bond-Lamberty et al., 2014; Chambers et al., 2016), although post-fire climate conditions are also an important control in other ecosystems (Shryock et al., 2015; Hansen et al., 2016). Seed limitation is mainly controlled by *in-situ* seed availability and seed dispersal, which are related to fire severity and size (Tautenhahn et al., 2016). Environmental filter often refers to local biophysical conditions (e.g., topographic positions, soil moisture, and solar radiation) and microclimate conditions that control seed germination and seedling establishment once seeds arrive. Post-fire seedling establishment is also vulnerable to drought and light conditions (Clark and St Clair, 2011; Walker et al., 2015; Harvey et al., 2016). The relative importance of seed limitation and environmental filtering on post-fire recovery varies depending on the interaction between species’ life-history traits and fire characteristics in the boreal forests. For example, in Alaskan black spruce (*Picea mariana*) forests, serotinious cones (the retention of seeds in cones that open after a fire) release abundant seeds after a fire, and environmental filtering is often the major factor that determines the post-fire successional trajectory due to the different regeneration abilities of deciduous hardwoods and conifers (Johnstone et al., 2010). In Central Siberian dark taiga forests dominated by *Abies sibirica, Picea obovata*, and *Pinus sibirica*, seed limitation plays a major role in determining post-fire successional trajectories via the different dispersal abilities of deciduous hardwoods and conifers (Tautenhahn et al., 2016). These studies have offered insightful implications for how an intensified fire regime will affect future ecosystem structures and functions in similar boreal forest ecosystems. However, relatively little is known about the mechanisms that regulate post-fire community assembly in the Siberian light taiga dominated by *Pinus sylvestris* and *Larix* spp, because the results from North American boreal forests may not be applicable to Siberian larch forests due to differences in species’ life history traits, fire regimes, climate, and environmental conditions. Furthermore, most of these studies are based on local observations and therefore are spatially and temporally limited (Johnstone et al., 2004; Lecomte et al., 2006; Cai et al., 2013). Consequently, its application over multiple wildfires and large regions remains uncertain.

Remote sensing offers a unique opportunity to explore burned patterns and the spatial controls on post-fire recovery over large spatial and temporal scales. Fire reduces the reflectance in the visible-to-near-infrared wavelengths (∼0.4–∼0.9 μm) due to the loss of photosynthetic vegetation and an accompanied increase in reflectance in short and middle infrared wavelengths (∼1.5–∼2.3 μm) due to char/ash accumulation on the ground (Lentile et al., 2006; Miller and Thode, 2007). Post-fire recovery has the opposite effect on surface reflectance due to the reestablishment of photosynthetic active vegetation. Such changes in spectral signatures, if well-calibrated with field survey data, can be valuable to characterize fire severity and vegetation recovery (Chu and Guo, 2014; Mouillot et al., 2014). For example, the normalized burn ratio (NBR) is often used as a fire severity indicator, due to its sensitivity to changes in vegetation and soil conditions (Miller et al., 2009). The normalized difference vegetation index (NDVI) and normalized difference shortwave infrared reflectance index (NDSWIR) are commonly used as indicators to monitor post-fire vegetation recovery in boreal forests (Cuevas-González et al., 2009) because they are associated with vegetation cover and canopy water content, respectively.

Siberian Larch forests constitute approximately 20% of global boreal forests and are associated with relatively low-severity fires due to their sparse stand structure in a permafrost environment (Conard et al., 2002). Chinese boreal larch forests are located on the southern border of Siberian larch forest and are sensitive to climate warming. Understanding the mechanisms that drive post-fire vegetation recovery could help to better predict potential responses of boreal biomes to climate warming and intensified wildfire regimes. In this research, we focused on a burned area that was disturbed in 2000, in the Great Xing’ an Mountains, China. We examined the effect of fire severity on an early post-fire vegetation recovery pattern using Landsat-derived time-series NDVI and NDSWIR as proxies of vegetation recovery. We also detected the roles of seed limitation and environmental filtering and their temporal dynamics during the post-fire forest successional stage. This study helps to understand the extended legacy effects of fire on Siberian larch forests, a globally important yet understudied ecosystem, and contributes to understanding the vulnerability of high-latitude ecosystems to environmental change by complementing results from North America (Johnstone et al., 2010; Kasischke et al., 2010).

## Materials and Methods

### Study Area

**FIGURE 1:**
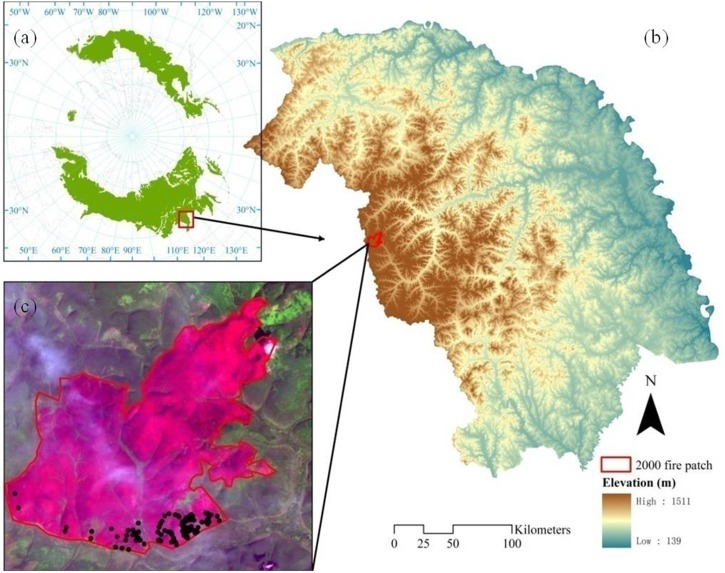
The location of the study area in circumpolar boreal forest ecosystem **(a)**, digitial elevation model **(b)**, and the burned scar shown in false color composite on the 2001 Landsat image (R:G:B = B7:B4:B1) **(c)**. Field survey sites were shown as solid black points in **c**.

The Huzhong National Natural Reserve is located in the Great Xing’ an Mountains of northeastern China (51°56′31″N 122°42′14″E to 51°17′42″N 123°18′05″E) and has a total area of 190,000 ha (**Figure 1**). The region is characterized by a continental monsoon climate with a mean annual precipitation and temperature of 500 mm and -4.7°C, respectively. The terrain is characterized by gently sloping uplands, and the valley bottom is frequently underlain by permafrost or seasonally frozen ground (Xu, 1998). Wildfire is a primary natural disturbance in this ecosystem, with a historical fire return interval of approximately 120 years (Xu, 1998). We selected an 8700-ha burned area in the Huzhong Natural Reserve, burned in 2000, to examine the relative influence of seed limitation and environmental filtering and their temporal dynamics in regulating post-fire forest recovery (**Figure 1**). This burned patch was selected because of its representativeness of Siberian larch forests and fire regime, minimum human intervention, and existence of field investigation of post-fire stand structure and function.

The area is representative of the Siberian light taiga—a closed canopy boreal forest dominated by *Betula platyphylla, Populus davidiana*, and *Populus suaveolens* during early-successional stages; and *Larix gmelinii*, and *P. sylvestris* var. *mongolica* in late-successional stages (Zhou, 1991). The fire regimes exhibit great spatial heterogeneity in occurrence, size and severity, primarily due to biophysical environments and associated fuel characteristics (Xu, 1998; Wu et al., 2013). Fires are relatively higher frequency and lower severity in well-drained, south-facing slopes where microclimates are relatively warm and dry and herbaceous species are dominant. Fires are relatively infrequent and more intensive on north slopes and terraces that are wet and cool and where two shrub species, *Ledum palustre* and *Vaccinium uliginosum*, are dominant. Post-fire tree seedlings mainly consist of larch and birch, and their density and composition are controlled by both fire severity and topographic positions (Cai et al., 2013).

### Overall Study Design

The aim of this study is to quantify the relative influence of seed limitation or environmental filtering in determining post-fire ecosystem recovery dynamics using field surveys and time series of remote sensing data. To do so, we used fire severity and distance to the nearest unburned forest, due to their relationship with *in-situ* residual mature trees and seed dispersal, as a proxy for seed availability. We used the topographic wetness index (TW) and soil radiation, due to their relationship with soil moisture and microclimate conditions, as proxies for environmental filters. We used time series of Landsat-derived vegetation indices as a proxy for post-fire forest recovery. To evaluate the suitability of each vegetation index in representing vegetation conditions, we compared field survey data with vegetation indices using the Pearson correlation. To evaluate whether fire severity and topographic conditions affect post-fire recovery dynamics, we compared the time series of vegetation indices under different fire severities and aspects using the paired-*t* test. Finally, to assess the relative influences of spatial controls on post-fire forest recovery, we used a machine learning approach to account for the potential complex relationship among post-fire vegetation recovery and a suite of variables.

### Datasets

#### Satellite Data and Preprocessing

Eleven Landsat 5 and 7 images (path 122, row 24^1^) acquired in peak growing seasons between 1999 and 2015 were used (**Table 1**). Most of the selected images were acquired in peak-summer months (June–September) with minimum cloud cover to minimize the influences of forest phenology on quantifying post-fire forest recovery dynamics (i.e., time-series NDVI and NDSWIR). The Landsat TM images acquired in 1999 and 2001 (**Table 1**) were chosen to calculate spectral fire severity (dNBR = NBR_2001_–NBR_1999_) (Miller and Thode, 2007; Miller et al., 2009).

**Table 1:** Time-series Landsat images for assessing post-fire recovery of boreal larch forest in the 2000 burned area.

ID	Acquisition data	Sensor	Year since fire
1	5/9/1999	TM7	Pre-fire
2	23/7/2001	TM5	1
3	30/8/2003	TM5	3
4	15/7/2004	TM5	4
5	25/6/2005	TM5	5
6	5/7/2006	TM5	6
7	20/8/2008	TM5	8
8	30/8/2009	TM5	9
9	26/8/2010	TM5	10
10	5/9/2011	TM5	11
11	6/7/2015	TM7	15


*No images in 2012 were found for the study area. All images are in the same path 122 and row 24.*

To ensure that the time series of vegetation indices reflected real changes in the state of the ecosystem, rather than phenology and senor-sun angle artifacts (the bidirectional reflectance distribution function), a radiometric correction was conducted to the selected Landsat images. The Landsat digital number (DN) was converted to top-of-atmosphere reflectance following a two-step process. First, the DNs were converted to radiance values using the bias and gain values specific for each scene. Second, the calculated radiance values were converted to top-of-atmosphere reflectance using the solar zenith angle and astronomical unit distance of the sun. We did not conduct a geometric correction because it had already been done by the USGS^2^.

### Field Survey Data

We selected 99 sites in the burned areas to sample the post-fire regeneration pattern of Chinese boreal larch forests (**Figure 1**) from 2010 to 2012. These sites were carefully selected to represent the variability of fire severity and environmental conditions. Within each site, a 50 m × 50 m plot was established based on its representativeness of fire severity, topographical position, and post-fire vegetation recovery pattern. Within each plot, the remaining unburned adult trees were counted for density and diameter in two subplots (10 m × 10 m), and post-fire recruited seedling/saplings for each species were surveyed for density in four subplots (5 m × 5 m). Detailed information regarding the field survey and forest recruitment conditions can be found in Cai et al. (2013) and Cai and Yang (2016). These field data were used for qualitative analysis of the NDVI and NDSWIR values.

### Proxy for Post-fire Forest Recovery: NDVI and NDSWIR

The images used for characterizing post-fire forest recovery were based on Landsat images acquired from 2001 to 2016. NDVI and NDSWIR were calculated using reflectance (ρ) values from the red (band 3), NIR (band 4), and SWIR (band 5) bands, as shown below:

$$\mathrm{NDVI}\;=\;(\rho_{\mathrm{NIR}}-\rho_{\mathrm{red}})/(\rho_{\mathrm{NIR}}+\rho_{\mathrm{red}})$$

$$\mathrm{NDSWIR}\;=\;(\rho_{\mathrm{NIR}}-\rho_{\mathrm{SWIR}})/(\rho_{\mathrm{NIR}}+\rho_{\mathrm{SWIR}})$$

### Proxy for Seed Limitation

Post-fire seed limitation for forest recruitment included *in-situ* residual mature trees and seed dispersal from around unburned areas. We used fire severity as a proxy for *in-situ* residual mature trees. The Landsat-derived differenced NBR (dNBR) is widely used for fire severity assessments in boreal forests (French et al., 2008; Soverel et al., 2011) and has been proven to be applicable in Chinese boreal forests (Fang and Yang, 2014). The dNBR has a high correlation with a field-based assessment of fire severity called the composition burn index (CBI) and can show variations in canopy tree mortality rate (Cocke et al., 2005). The dNBR was computed from pre-fire (09/05/1999) and post-fire (09/14/2000) images using the algorithm described by Lentile et al. (2006). As the effective seed dispersal distance of the dominant species Gmelin’s larch is approximately 100 m, we used a moving window (5 × 5 pixels) resampling approach to minimize the neighborhood effect of nearby surviving trees on post-fire forest recovery.

In this analysis, pre-fire species composition and age retrieved from forest inventory data were employed as proxy data for seed availability. Information regarding pre-fire vegetation type was needed for comparing the responses of different forest types to fire disturbance. The Forest Inventory Data conducted in 1990 is the available land-cover map before the 2000 fire. These data were used to identify the pre-fire forest type of the burned area. We also calculated the proportion of broadleaf species, which was used as an independent factor in regulating post-fire forest recovery. We used ArcGIS to calculate the distances to the nearest unburned area as a proxy for seed availability and delivery from around the unburned area (Greene et al., 1999).

### Proxy for Environmental Filters

We calculated a TW from a digital elevation model using the Hydrology module in ArcGIS to indicate soil moisture availability due to its strong control on tree regeneration and establishment ability (Zinko et al., 2005). We also calculated a solar radiation index to indicate the potential atmospheric water demand (Kumar et al., 1997). The solar radiation index was estimated using the Solar Radiation analysis toolset in ArcGIS. The toolset used variability in the orientation (slope and aspect) to calculate direct and diffuse radiation for each pixel of the elevation model using viewshed algorithms. We report total insolation during the growing season for each pixel. Slope and aspect were also included. All of the indices were calculated from a 30 m resolution digital elevation model.

### Data Analysis

To explore the suitability of each vegetation index in representing post-fire forest recovery, we compared field survey data (tree recruitment density and species composition) with NDVI and NDSWIR. The Pearson correlation coefficient was calculated in addition to NDVI, NDSWIR, log-transformed tree recruitment densities and tree species composition. These vegetation indices were calculated from TM images that were acquired on August 26, 2010.

To explore post-fire forest recovery trajectories, time series of NDVI and NDSWIR were sampled. First, the minimum distance between each sampling point was set as 1000 m to remove a spatial autocorrelation issue. Second, a normalized time series vegetation index was calculated to remove the potential bias caused by phonology, the senor artifact, or topographic influence among years. To do so, we created a 2000 m buffer zone around the burned patch, and classified the landscape (burned patch + buffer zone) into three land types based on topographic position because of its influence on ecosystem and fire characteristics (flat valley bottom, south-facing, and north-facing slopes; see *study area* description). In each land type, the mean value of the vegetation index of buffer zone (termed as unburned area, e.g., NDVI_unburned_) and burned patches (termed as burned area, e.g., NDVI_burned_) was calculated. The normalized NDVI and NDSWIR in each land type were calculated using the following formulas:

$$\mathrm{Normalized}\;\mathrm{NDVI}\;=\;({\mathrm{NDVI}}_{\mathrm{burned}}-{\mathrm{NDVI}}_{\mathrm{unburned}})/{\mathrm{NDVI}}_{\mathrm{unburned}}$$

$$\mathrm{Normalized}\;\mathrm{NDSWIR}\;=\;({\mathrm{NDSWIR}}_{\mathrm{burned}}-{\mathrm{NDSWIR}}_{\mathrm{unburned}})/{\mathrm{NDSWIR}}_{\mathrm{unburned}}$$

The mean and standardized values of vegetation indices were plotted against time since last fire to show the post-fire forest recovery trajectory.

A paired *t*-test was applied to examine the variations in NDVI and NDSWIR between high severity and low severity burned areas, as well as among different topographic positions (flat valley bottoms, south-facing, and north-facing slopes). Statistical significance in these analyses was assessed at *a* = 0.05.

We also used a boosted regression tree (BRT) analysis to examine the influence of a suite of explanatory variables, including fire severity, TW, solar radiation, distance to the nearest unburned area and pre-fire forest type, on post-fire forest recovery. BRT is a machine-learning approach based on classification and regression trees (Elith et al., 2008). It is capable of handling complex nonlinear relationships and missing values without the restrictive assumptions of parametric statistics. The relationship between dependent and independent variables could visually be shown using a partial dependency plot, and it could calculate the relative influence of each independent variable on the dependent variable. The BRT method combines the advantages of ensemble regression trees (e.g., random forest), which relate a response to their predictors by recursive binary splits and boosting algorithms, which combine many simple models to give improved predictive performance (Elith et al., 2008). In the fitted BRT models, both NDVI and NDSWIR were selected as response variables. Biotic and abiotic explanatory variables for the two analytical approaches are listed in **Table 2**. The BRT models were fitted with the following parameters: Gaussian error distribution, a learning rate of 0.005, and a bag fraction of 0.5. Others used default parameters. The BRT analysis was run in the *gbm* module of *R* software (R Core Team, 2013).

**Table 2:** List of dependent and predictor variables considered in Boosted regression tree analysis.

Variable		Units	Description
Dependent variable	Yearly NDSWIR	NDSWIR	Continuous variable, a proxy for post-fire vegetation recovery status, 2–15-year post-fire
Seed availability	dNBR	dNBR	Continuous variable, a proxy for fire severity
	Distance to the nearest unburned area	m	Continuous variable, a proxy for seed availability from unburned area
Environmental condition	Solar radiation		Continuous variable, calculated using aspect and slope, a proxy for light conditions
	Topographic wetness index		Continuous variable, calculated using elevation, aspect and slope, a proxy for site moisture condition


## Results

**FIGURE 2:**
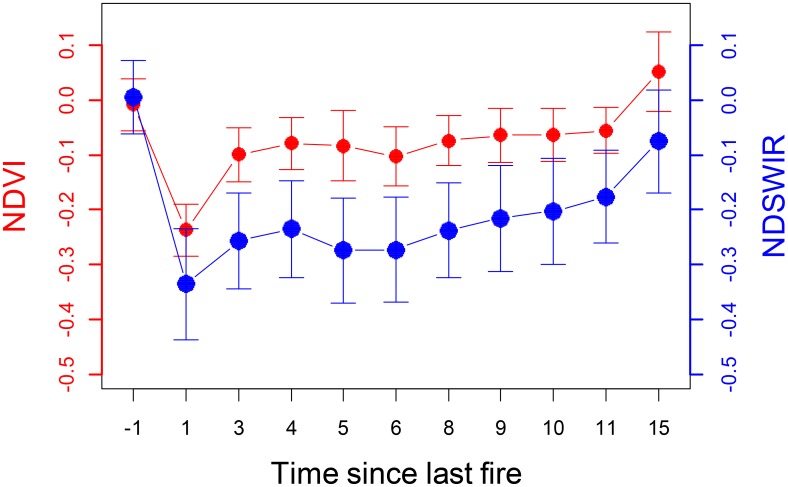
The normalized difference of Normalized Difference Vegetation Index (red line) and Normalized Difference Shortwave Infrared reflectance Index (blue line) between burned areas and unburned control sites plotted as a function of time since last fire.

The normalized NDVI and NDSWIR removed the influence of interannual variability in climate, and its dynamics reflected the post-fire vegetation recovery dynamics to fire severity and environmental conditions. A negative value of these indices indicated departure from pre-fire vegetation conditions. **Figure 2** shows reduced values 1 year after the wildfire, followed by a gradual recovery period. It took approximately 10 years for NDVI to recover to the pre-fire state, and at least 15 years for NDSWIR (**Figure 2**). Our results showed that different vegetation indices followed special recovery trajectories, reflecting the fact that they may represent different aspects of ecosystem characteristics.

**FIGURE 3:**
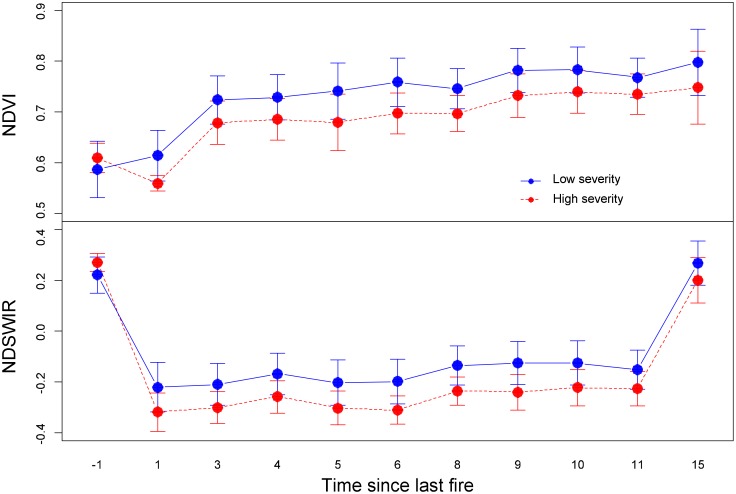
Post-fire dynamics of NDVI and NDSWIR values (mean + SD) in different fire severity levels.

**FIGURE 4:**
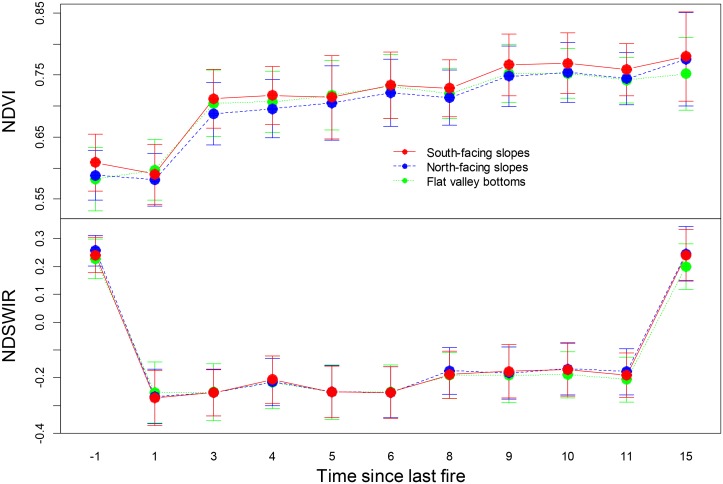
Post-fire dynamics of NDVI and NDSWIR values (mean + SD) in different topographic positions.

Post-fire recovery rates also depended on the degree to which the larch forest ecosystem had changed due to the fire disturbance. **Figure 3** shows the variation in vegetation recovery between high severity and low severity burned areas. The recovery rate was higher in the low severity burned area (**Figure 3**). A high fire severity burn could exert a long-term negative effect on vegetation recovery. Our field observations showed that post-fire tree recruitment was different among topographic positions, with south-facing slopes favoring broadleaf tree recruitment. Our results also showed that the recovery rate of NDVI and NDSWIR did not differ significantly (*p* > 0.05) for different topographic conditions (**Figure 4**).

**FIGURE 5:**
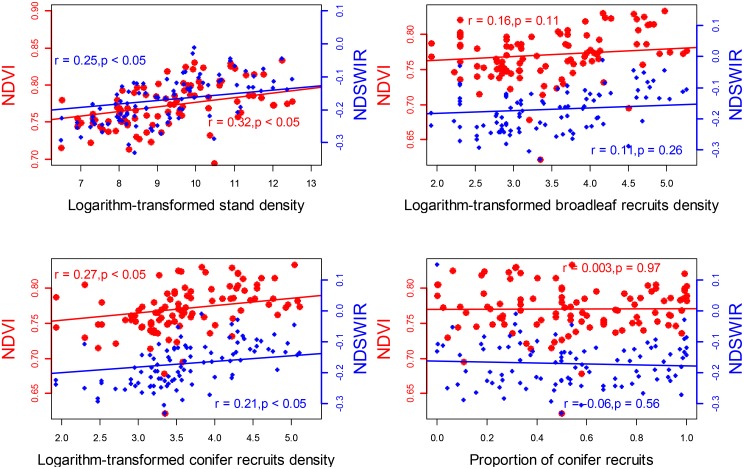
The relationship between field surveyed post-fire tree recruitment and NDVI and NDSWIR values (10 years after fire), respectively.

To analyze whether NDVI and NDSWIR could reflect post-fire vegetation recovery conditions, we compared the vegetation indices with field sampling data. The results showed that NDVI and NDSWIR had a positive relationship with post-fire stand density (*r* = 0.32 and 0.25 for stand density, respectively, *p* < 0.05) but had no relationship with post-fire forest composition (*p* > 0.05) (**Figure 5**).

**FIGURE 6:**
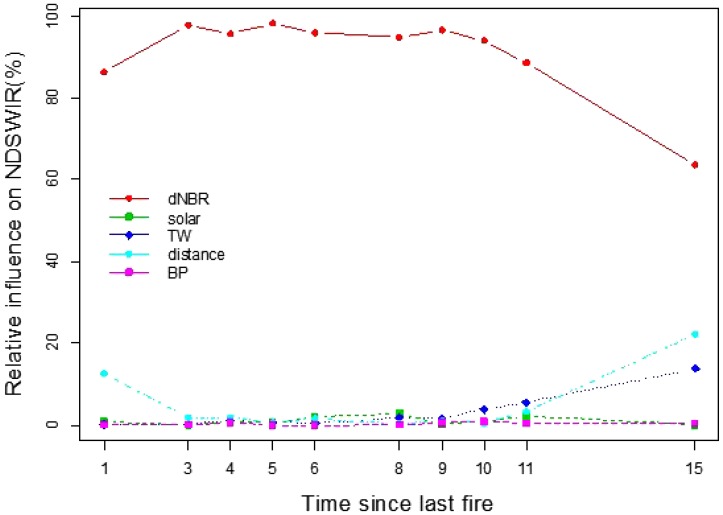
Relative importance of predictor variables on NDSWIR. dNBR stands for differenced Normalized Burn Ratio; solar stands for solar radiation; TW stands for topographic wetness index; distance stands for distance away from unburned area; BP stands for pre-fire proportion of broadleaf species.

The BRT analysis showed the relative contributions of fire severity and other independent variables (**Table 2**) to vegetation recovery. The results showed that fire severity was the most important factor in determining post-fire NDVI and NDSWIR recovery (**Figure 6**). It explained more than 60% variation of post-fire vegetation recovery. Pre-fire forest structure and distance to the nearest unburned area had little influence on forest recovery. Although solar radiation and TW, which depict environmental conditions, had relatively small effects on NDVI and NDSWIR, their effects increased with forest succession. Because of the similar trend between NDVI and NDSWIR, only the relative influence of each predictor on NDSWIR is shown in **Figure 6**.

## Discussion

### The Suitability of NDVI/NDSWIR in Monitoring Post-fire Forest Recovery

Our results, consistent with other studies (Cuevas-González et al., 2009; Jones et al., 2013; Meng et al., 2015; Chu et al., 2016, 2017), suggested that time series of spectral indices are an effective way to monitor post-fire vegetation recovery dynamics over large spatiotemporal scales. The NDVI, a commonly used vegetation greenness index, is often used to assess chlorophyll abundance and the vigor of green vegetation. The NDSWIR, a shortwave infrared-based index, is sensitive to canopy water content and stand structure. Therefore, these two spectral indices indicate different aspects of vegetation characteristics. In this analysis, NDVI reached its maximum quickly after the fire and resulted in a faster vegetation recovery rate, suggestive of rapid recruitment of shrubs and herbaceous vegetation after fire (Hart and Chen, 2006). However, NDSWIR recovered at slower rate, indicative of much slower establishment and growth of forest stands. Although NDSWIR and NDVI are good indicators of vegetation cover and structure, they offer little information regarding species composition (**Figure 5**). Thus, they should be used with caution when applying spectral indices for detecting the legacy effects of disturbance on post-fire forest successional trajectories (i.e., dynamics of vegetation communities). Nevertheless, spatially and temporally consistent time series of spectral indices remain an effective way to assess post-fire recovery and forest resilience in a warmer climate and intensified fire regime.

### Time Needed for Forest Recovery

Quantifying the time needed for forest recovery is useful for evaluating the long-term legacy effects of disturbances to ecosystem properties. However, this analysis also suggested that rate of forest recovery is dependent on vegetation indices (i.e., NDVI and NDSWIR) and ecosystem types. It took at least 10 years for NDVI to return to control sites, and it would be longer for NDSWIR (**Figure 2**). This recovery time is similar to the recovery rate derived by Cuevas-González et al. (2009) in central Siberia. However, it is longer than that in North America and Canada, which are less than 8 years (e.g., Hicke et al., 2003; Goetz et al., 2006). These variations might be attributed to many aspects such as differences in fire regime, community composition and tree traits (e.g., fire adaptive trait) between North American and Siberian boreal forests. For example, black spruce [*Picea mariana* (Mill.) B.S.P.], a dominant species in many North American boreal forests, is serotinous and could release abundant seeds immediately after a fire (Johnstone et al., 2008) and therefore experience a faster recovery than boreal larch forests.

The rapid increase in NDVI following fire was considered to reflect the restoration of vegetation cover (including tree, shrub, and herbaceous vegetation). Forest regeneration and the quick recovery of vegetation cover are assumed to occur in the first few years after a fire, and this community structure could persist for a long time (Johnstone et al., 2004; Alexander et al., 2012). The rapid recovery in NDVI showed that vegetation cover can likely reach a pre-fire state within 10 years in the Great Xing’ an Mountains (**Figure 2**). However, NDVI could not identify species composition. The relatively slow recovery of NDSWIR indicated that the post-fire forest structure could not recover in a short time. As NDVI is assumed to have a positive relationship with forest NPP (Turner et al., 2004), the rapid increase in NDVI indicated a high resilience of NPP to wildfire. Our result indicated that ecosystem function is less sensitive to disturbance than forest structure (Cai and Yang, 2016). Boreal larch forests are quite resilient to fire disturbances, showing quick recovery, at least in terms of rapid return to previous states of vegetation cover not only in low-severity burned stands but also in high-severity burned stands.

### Seed Availability vs. Environmental Filtering

The dNBR was frequently used for quantifying the conditions of affected vegetation after fire (Goetz et al., 2006; Meng et al., 2015; Bartels et al., 2016). Fire severity (dNBR) could reflect information about post-fire survival trees and residual organic layer depth, which control seed availability and seed bed quality for tree regeneration. In the Great Xing’ an Mountains, the organic layer in unburned areas is relatively shallow compared to that in North American boreal forests, and it is consumed by relatively low severity fires (based on field observations). The difference in fire severity is often considered as the variation in *in-situ* survival trees. Severe fires significantly reduced forest recovery rates, and this negative effect on post-fire forest recovery would persistently dominate for at least 10 years. This result is convincing evidence that fire severity has a legacy effect on forest recovery, confirming previous studies (Pan et al., 2011; Fletcher et al., 2014; Brown et al., 2015). Seed availability is the most important factor in regulating post-fire recovery trends. However, distance to the nearest unburned area had a relatively small effect. Seed dispersal from unburned area contributed little to post-fire forest recovery. This might connect with the fact that the effective seed dispersal distance of the dominant conifer species (*L. gmelinii*) is less than 100 m. Mature trees within the burned patch have similar influences on tree regeneration as the edge of the burned patch. Over the expanse of the burned area, post-fire tree regeneration is dependent on *in-situ* residual seed banks or survival propagules.

Environmental factors had a relatively small influence on post-fire forest recovery. However, their effect increased with succession. This result indicated that environmental filtering would mitigate the legacy effect of fire disturbance on forest ecosystems. The legacy effects caused by spatial heterogeneities of fire severity tend to become mitigated with succession in terms of NDVI and NDSWIR (**Figure 3**). However, compared to upland sites, in valley bottoms, differences in NDVI/NDSWIR between fire severity patches are more persistent. This result is likely due to the existence of seasonal permafrost and poor nutrient conditions in valley bottoms, where Gemlin’s larch is adaptive.

Our results showed that elevation changes also influence post-fire vegetation recovery, likely due to its relationship to climatic conditions. This result indicated that climatic conditions are also the drivers of vegetation response after disturbances. Post-fire climate might control tree regeneration and interact with factors such as seed availability and environmental conditions to alter post-fire recovery (Meng et al., 2015). Post-fire climate in the first growing seasons following fire may be highly important for forest recruitment (Shryock et al., 2015). As we only assessed post-fire forest recovery in a single burned patch, climate factors were not considered comprehensively in this study. Additionally, climate should be considered in studies with multiple fires.

The BRT models could significantly explain the temporal and spatial variation in NDVI and NDSWIR as a function of independents indicating seed availability and environment properties. Our field sampling data showed that NDVI, especially NDSWIR, has a good positive relationship with tree recruitment density. Hence, the influence of selected independent variables on NDVI/NDSWIR could reflect underlying ecological processes that affect post-fire vegetation recovery. Seed availability and environmental filtering are two important processes that control post-fire forest dynamics. Seed availability had a dominant effect during the early successional stage, but its effect was mitigated by environmental filtering.

### Limitations

First, we analyzed the relative influence of seed limitation and environmental filtering on post-fire vegetation recovery for only one fire, which may not fully represent the full spectrum in terms of fire severity and environmental gradients for larch forest. However, this analysis also provided us to minimize other confounding factors such as past disturbance history, climate variability, and vegetation conditions, and therefore allowed us to confidently assess the effects of seed limitation and environmental filtering on post-fire recovery. Second, although time series analyses of NDVI and NDSWIR are good indicators of photosynthetically active vegetation or “greenness,” they did not necessarily reflect recovery in other important ecosystem variables such as biogeochemistry, radiation budgets, or water balance. In the future, we will utilize multiple recovery indicators to provide complementary measurements of vegetation dynamics across many fires to study the interactions among ecosystem vulnerability, disturbances, and climate in high-latitude ecosystems.

## Implications

The post-fire forest recovery rate is strongly related to ecosystem carbon storage and its resultant effects on climate feedback. Slow vegetation recovery rates likely induce low carbon storage. Post-fire ecosystem carbon storage shows a sigmoid pattern that initially decreases and then increases, and eventually balances (Yang et al., unpublished). As fire disturbances in boreal forests are predicted to be larger and more severe, these changes imply an increasing risk of seed limitation for regulating post-fire forest recovery, loss of ecosystem resilience, and increases in carbon release. The coupled effect of vegetation destruction and low forest recovery rates due to high-severity wildfires inevitably leads to a rapid increase in carbon loss.

Using spatially and temporal consistent spectral indices, we showed that fire severity is a dominant factor in post-fire forest recovery, and its effect could last many decades. The effect of environmental filtering on forest recovery would increase with succession, reflecting the convergence of ecosystem to climax regardless of the initial conditions. *In-situ* seed availability remains the primary factor in regulating vegetation recovery. Understanding the mechanisms that underlie the interaction of fire disturbance and environmental conditions on vegetation recovery will assist post-fire forest management efforts to enhance the resilience of ecosystems in Siberian larch forests.

## Author Contributions

ZL, JY, and WC conceived and designed research. WC performed the experiments, analyzed the data and wrote the manuscript. ZL and JY revised the manuscript. YY helped in conducting the experiments, analyzing the data and editing the manuscript.

## Conflict of Interest Statement

The authors declare that the research was conducted in the absence of any commercial or financial relationships that could be construed as a potential conflict of interest.

## Footnotes

1 http://glovis.usgs.gov/

2 http://landsat.usgs.gov/
